# PTX3 Intercepts Vascular Inflammation in Systemic Immune-Mediated Diseases

**DOI:** 10.3389/fimmu.2019.01135

**Published:** 2019-05-29

**Authors:** Giuseppe A. Ramirez, Patrizia Rovere-Querini, Miriam Blasi, Silvia Sartorelli, Maria Chiara Di Chio, Mattia Baldini, Rebecca De Lorenzo, Enrica P. Bozzolo, Roberto Leone, Alberto Mantovani, Angelo A. Manfredi, Enrico Tombetti

**Affiliations:** ^1^Università Vita-Salute San Raffaele, Milan, Italy; ^2^Unit of Immunology, Rheumatology, Allergy and Rare Diseases, IRCCS Ospedale San Raffaele, Milan, Italy; ^3^Division of Immunology, Transplantation and Infectious Immunity, IRCCS Ospedale San Raffaele, Milan, Italy; ^4^Humanitas Research Center - IRCCS, Rozzano, Italy; ^5^Humanitas University, Rozzano, Italy; ^6^The William Harvey Research Institute, Queen Mary University of London, London, United Kingdom

**Keywords:** PTX3, autoimmunity, lupus, rheumatoid arthritis, Takayasu arteritis, giant cell arteritis, ANCA associated small vessel vasculitis, intravascular immunity

## Abstract

PTX3 is a prototypic soluble pattern recognition receptor, expressed at sites of inflammation and involved in regulation of the tissue homeostasis. PTX3 systemic levels increase in many (but not all) immune-mediated inflammatory conditions. Research on PTX3 as a biomarker has so far focused on single diseases. Here, we performed a multi-group comparative study with the aim of identifying clinical and pathophysiological phenotypes associated with PTX3 release. PTX3 concentration was measured by ELISA in the plasma of 366 subjects, including 96 patients with giant cell arteritis (GCA), 42 with Takayasu's arteritis (TA), 10 with polymyalgia rheumatica (PMR), 63 with ANCA-associated systemic small vessel vasculitides (AAV), 55 with systemic lupus erythematosus (SLE), 21 with rheumatoid arthritis (RA) and 79 healthy controls (HC). Patients with SLE, AAV, TA and GCA, but not patients with RA and PMR, had higher PTX3 levels than HC. PTX3 concentration correlated with disease activity, acute phase reactants and prednisone dose. It was higher in females, in patients with recent-onset disease and in those with previous or current active vasculitis at univariate analysis. Active small- or large- vessel vasculitis were the main independent variables influencing PTX3 levels at multivariate analysis. High levels of PTX3 in the blood can contribute to identify an increased risk of vascular involvement in patients with systemic immune-mediated diseases.

## Introduction

Vascular inflammation reflects the dynamic interaction of circulating cells, blood molecules and vascular structures which plays a role in vascular homeostasis and in systemic or tissue/organ-limited autoimmunity (1–5). The vasculature recruits cellular and immune effectors to facilitate the intercellular signaling required to deploy an inflammatory and immune response (5, 6). Vessels are frequently targeted in immune mediated-diseases, although in a subset of patients vascular inflammation is prominent, resulting in overt vasculitis.

Pentraxins are part of an ancestral humoral innate network, evolutionarily rooted before the divergence of the immune and the haemolymphatic system. Pentraxin-3 (PTX3), a member of the long-pentraxin family, has changed little during the evolution, likely due to its role in multiple biological events (7, 8). In contrast to other pentraxins such as C-reactive protein (CRP), PTX3 is mostly generated at sites of inflammation rather than as a consequence of centralized hepatic synthesis. Neutrophils massively release PTX3 upon activation, while endothelial cells or macrophages synthetize the molecule, sustaining PTX3 production for longer times (9–11). In the extracellular space, PTX3 opsonizes self and foreign antigens and contributes to the structural and functional fitness of the extracellular matrix. Evidence has been acquired for a potential pathogenic role of PTX3 in a broad range of events from host defense to fertility, cancer biology, autoimmunity, regulation of angiogenesis and tissue repair (7, 12–19).

Enhanced expression of PTX3 has been reported in multiple systemic autoimmune diseases (20–27). Given that most cellular sources of PTX3 can be involved in vascular inflammation, and that PTX3 has been shown to be specifically involved in the regulation of the cross talk between the main players of intravascular immunity, including neutrophils, apoptotic cells, platelets, endothelial and antigen presenting cells (22, 28, 29), we undertook an observational study assessing systemic expression of PTX3 in healthy subjects and multiple inflammatory diseases with variable vascular involvement.

## Patients and Methods

Upon written informed consent, 366 subjects followed up at San Raffaele University Hospital, Milan, Italy were recruited including: 96 patients with GCA, 42 with TA, 10 with PMR, 38 with granulomatosis with polyangiitis (GPA), 15 with eosinophilic granulomatosis with polyangiitis (EGPA), 10 with microscopic polyangiitis (MPA), 55 with systemic lupus erythematosus (SLE), and 21 with rheumatoid arthritis (RA). Seventy-nine healthy volunteers served as controls. All patients gave their written informed consent for participation in this study (Autoimmuno-Mol protocol, approved by the Ethics Committee of the San Raffaele Institute, Milan, Italy; reference number 2/2013/INT). Patients were classified according to the 1990 American College of Rheumatology (ACR) classification criteria for GCA and PMR (30), the 1996 Sharma's diagnostic criteria for TA (31), the European Medicine Agency algorithm for classification of GPA, EGPA and MPA (32), the revised 1997 ACR or the 2012 SLE International Collaborating Clinics (SLICC) classification criteria for SLE (33, 34) and the 2010 ACR/European League Against Rheumatism(EULAR) classification criteria for RA (35).

Basic demographics (including gender, age at sampling and disease duration), disease activity and accrued irreversible damage, dose of prednisone or equivalents, erythrocyte sedimentation rate (ESR) and CRP values at time of sampling were recorded. In patients with SLE, complement levels and anti-DNA titres were collected. Disease activity for group comparison was quantitated by employing a 28-joint disease activity score (DAS-28) for RA, the SLE disease activity index 2000 version (SLEDAI-2K) for SLE (36), the Birmingham Vasculitis Activity Score version 3 (BVAS v3) for anti-neutrophil cytoplasm antibody (ANCA)-associated vasculitides (AAV) (37) and the Indian Takayasu Activity Score for TA (ITAS2010) (38). In patients with SLE, disease activity was also estimated by employing the British Isles Lupus Assessment Group (BILAG) 2004 version index (39) and a 0.0-3.0 physician global assessment scale (PGA). A 0-10 visual analog scale (VAS) also measured SLE patients' impression about their global health status. Organ damage was determined by the SLICC/ACR Damage Index (SDI) for SLE (40), the Vasculitis Damage Index (VDI) for AAV and GCA (41) and the TA Damage Score (TADS) for TA (42). Disease activity and damage scores were made homogeneous by calculating Z-scores (i.e., $\frac{x-mean}{standard\;deviation}$) for activity and damage (Z-activity and Z-damage). In parallel to quantitative assessment, a binary evaluation of disease activity and damage was performed. The former was based on the Physician Global Assessment of disease avidity (Inactive vs. Active/smoldering), the latter by the presence vs. absence of items related to vasculitic manifestations in the abovementioned scores. In patients with SLE, lupus chilblains, skin/digital vasculitis/ischemia, urticarial vasculitis, gastrointestinal vasculitis, choroidopathy or retinal vasculitis, cerebral vasculitis and alveolar hemorrhage were considered as relevant vasculitic manifestation, whereas Raynaud's phenomenon was not. In patients with AAV, “pure” vasculitic manifestations included absence of ear-nose-throat (ENT) or orbital involvement and presence of purpura, scleritis, episcleritis, optic neuritis, renal involvement, peripheral neuropathy, hemorrhagic alveolitis, or diagnosis of MPA.

PTX3 plasma levels were measured by a sandwich ELISA based on original reagents developed in house. 96 well plates (Nunc MaxiSorp cat. 446612) were coated with 100 μl anti-hPTX3 monoclonal antibody (MNB4 (43) 1 μg/ml−100 ng/well) in coating buffer (15 mM carbonate buffer pH 9.6) and incubated overnight at 4°C. Plates were washed after each step with 300 μl/well of washing buffer (PBS 1X with Ca^++^ Mg^++^ + 0.05% Tween 20, pH 7.00). After coating, non-specific binding to the plates was blocked with 5% dry milk in washing buffer (2 h at room temperature), then 50 μl in duplicate of recombinant human PTX3 standard (from 75 pg/ml to 2.4 ng/ml) and human plasma (diluted in PBS 1X w/o Ca^++^ Mg^++^ + 2% BSA, pH 7.00), were plated. 1 μl of 2.5% Polybrene was added to 50 μl of plasma and incubated at room temperature for 10 min before dilution. After 2 h at 37°C, plates were incubated with 100 μl /well of purified and biotinylated rabbit IgG anti hPTX3 (5 ng/well) in washing buffer (1 h at room temperature), followed by incubation with 100 μl/well-streptavidin conjugated to horseradish peroxidase (cat. SB01-61, Biospa, Milan, Italy) diluted 1:2,000 in washing buffer (1 h at room temperature). Finally, 100 μl of 1-Step™ Ultra TMB-ELISA Substrate Solution (cat. 34029, Thermo Scientific, Rockford, IL, USA) were added and the reaction was blocked after 10 min with 50 μl of 2M Sulphuric Acid (H_2_SO_4_) before reading the plates at 450 nm in an automatic ELISA reader. All the procedure was performed by personnel blind to patients' characteristics. For each biological sample, 2 dilutions in duplicate wells were evaluated and mean PTX3 content was calculated converting Abs450 values to protein concentration by means of the standard curve with recombinant purified hPTX3. Analysis was performed with SoftMax Pro software v5.3 (MDS Analytical Technologies, USA) and linear regression was used to interpolate unknown samples. Lower limit of detection of the assay was 75 pg/ml, interassay variability was from 8 to 10%; no cross reaction was observed with short pentraxins CRP and SAP.

PTX3 was measured in four different batches. Inter-batch variability was corrected by normalization based on HC samples. The relative frequencies of laboratory and clinical categorical variables were compared by using chi-square test with Fisher's exact correction as appropriate. Quantitative variables were compared by using Spearman's correlation tests. Differences in quantitative variables among groups were assessed by employing Mann-Whitney U-test or Kruskal-Wallis' test for multiple comparisons. We also employed generalized linear models with gamma distribution of the dependent variables and log function as a link function to assess the effect of each quantitative or qualitative variable on PTX3 levels. Data were processed and analyzed by employing Microsoft Excel^®^ 2013 and IBM SPSS^®^ version 15-23. Data are expressed as median (interquartile range, IQR) unless otherwise specified.

## Results

### Expression of PTX3 in Systemic Autoimmune Diseases

We enrolled 287 patients diagnosed with systemic autoimmune diseases. Seventy-nine volunteers served as controls. Twenty-one patients had RA, a systemic inflammatory disease targeting the synovial membrane, 55 had SLE, the prototypic systemic autoimmune disease, including 19 patients with vasculitic features, ten had PMR, a fruste form of GCA with prominent osteoarticular inflammation sparing the vasculature. Moreover, we enrolled patients with primary systemic vasculitis of small vessels (*n* = 63, including 38 with GPA, 15 with EGPA) and large vessels (*n* = 138, including 96 with GCA and 42 with TA). Table 1 summarizes patients' main characteristics.

**Table 1:** General features of patients included in the study.

	**RA (*n* = 21)**	**SLE (*n* = 55)**	**PMR (*n* = 10)**	**AAV (*n* = 63)**	**GCA (*n* = 96)**	**TA (*n* = 42)**
Age at diagnosis (year)	ND	26.2 (18.6- 34.2)	72.0 (68.5- 73)	48.5 (36.5 - 61.5)	74.0 (67.0 - 77.0)	30.0 (24.0 - 40.5)
Age at sampling (years)	63.0 (43.0- 67.0)	38.2 (31.2- 48.9)	73.0 (69.3- 77)	59.5 (47.8 - 67.9)	75.2 (69.4 - 79.5)	46.0 (35 - 53)
ESR (mm/h)	16.5 (12.8 - 23.5)	20.0 (7.0 - 38.5)	30.0 (20.0 - 50.0)	13.0 (6.5- 31.5)	31.0 (18.0 - 57.8)	15 (7.8- 30.3)
CRP (mg/l)	3.5 (2 - 6.7)	2.1 (0.3 - 6.1)	10.4 (8.7 - 22.3)	0.8 (0.23 - 6.2)	11.0 (2.26 - 31.4)	2.25 (1.03 - 7.3)
PDN dose at sampling (mg/day)	5.0 (2.5 - 5)	3.8 (0.0 - 5.0)	1.9 (0.0 - 17.2)	5.0 (5.0 - 6.5)	5.0 (0.0 - 12.5)	5.0 (0.0 - 5.0)
Z-activity	−0.42 (−0.47 - −0.28)	−0.33 (−0.56 - 0.35)	NA	−0.4 (−0.4- −0.07)	−0.23 (−0.23 - −0.07)	−0.42 (−0.47 - −0.28)
Z-damage	ND	−0.2 (−0.2 - −0.07)	NA	−0.06 (−0.06 - 0.01)	−0.14 (−0.14 - −0.06)	−0.12 (−0.57 - 0.19)

**Figure 1:**
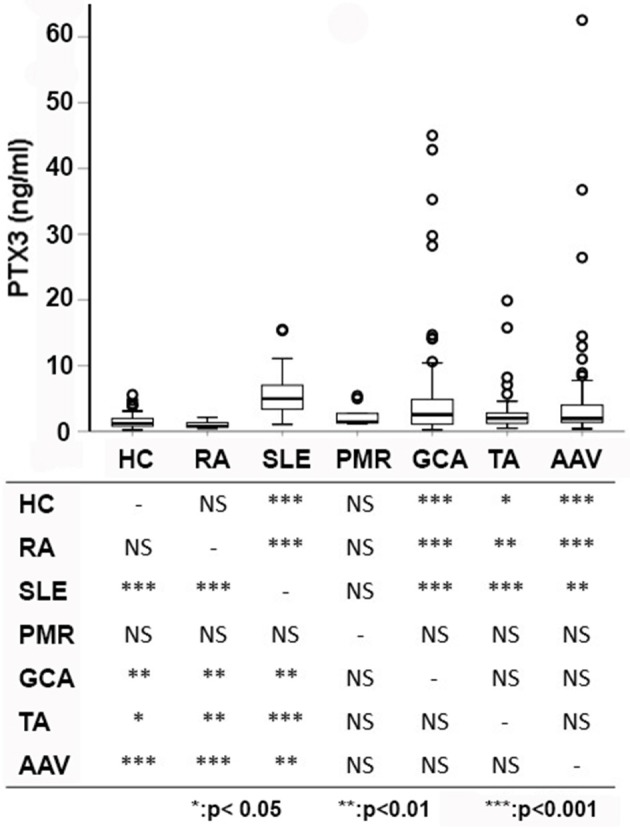
PTX3 levels among different diseases. PTX3 plasma levels were compared among patients with multiple distinct autoimmune diseases and HC. Pairwise comparisons among groups are reported below. Patients with PMR and RA did not differ from HC, whereas patients with other immune-mediated diseases showed significantly higher levels of PTX3 compared to patients with RA and HC. Patients with SLE had higher levels of PTX3 compared to patients with large- (TA, GCA) and small-vessel vasculitides (AAV). AAV, ANCA-associated vasculitides; GCA, giant cell arteritis; HC, healthy controls; PMR, polymyalgia rheumatica; RA, rheumatoid arthritis; SLE, systemic lupus erythematosus; TA, Takayasu's arteritis; ^*^p < 0.05; ^**^p < 0.01; ^***^p < 0.001.

PTX3 levels were significantly higher in patients with systemic inflammatory immune-mediated and autoimmune diseases (2.33 ng/ml, IQR: 1.26–4.89, *n* = 287) than in HCs (1.22 ng/ml, IQR:0.80–1.98, *n* = 79; *p* < 0.001). However, despite often comparable levels of systemic inflammation, PTX3 expression was heterogeneous in patients with different diseases. RA and PMR had plasma PTX3 levels comparable to those of HC. In contrast, PTX3 plasma levels of patients with SLE, AAV, GCA, and TA were significantly higher. Moreover, patients with SLE had higher levels of PTX3 compared to patients with AAV, GCA and TA (p < 0.001 for AAV and TA, *p* = 0.001 for GCA; Figure 1).

### PTX3 Correlates With Disease Activity but Not With Accrued Damage

**Figure 2:**
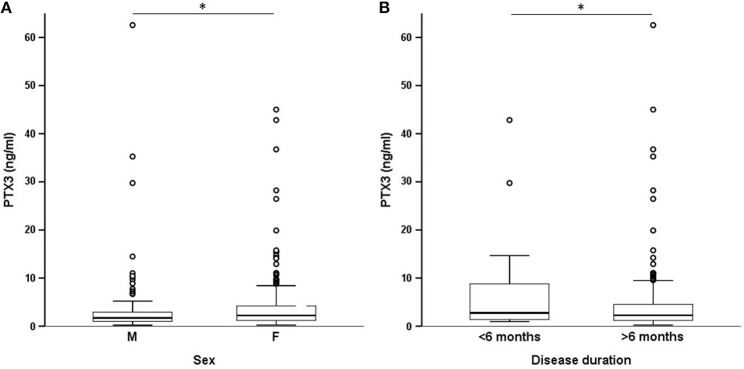
PTX3 levels and demographics. These panels show the existing differences in systemic expression of PTX3 by selected demographics. PTX3 levels were higher in females **(A)** and in patients with early-onset disease **(B)**. ^*^p < 0.05.

**Figure 3:**
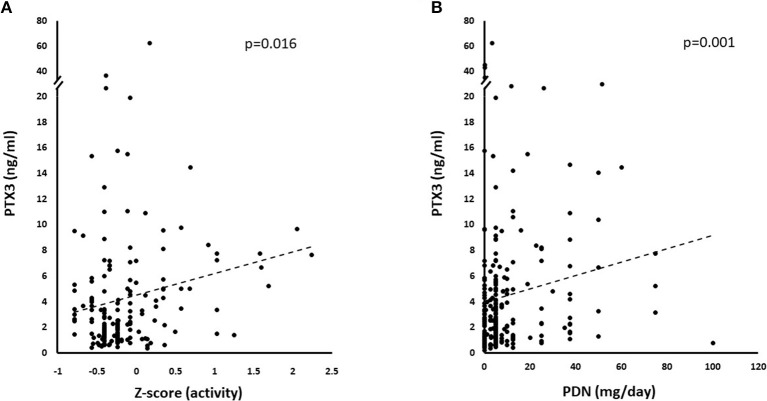
Correlations with PTX3 levels. PTX3 correlated with multiple disease and treatment-related variables at univariate analysis. **(A)** Depicts the linkage between increasing normalized activity score (Z-activity) and PTX3 plasma levels. **(B)** Shows the potential influence of corticosteroid treatment on PTX3 circulating levels.

**Figure 4:**
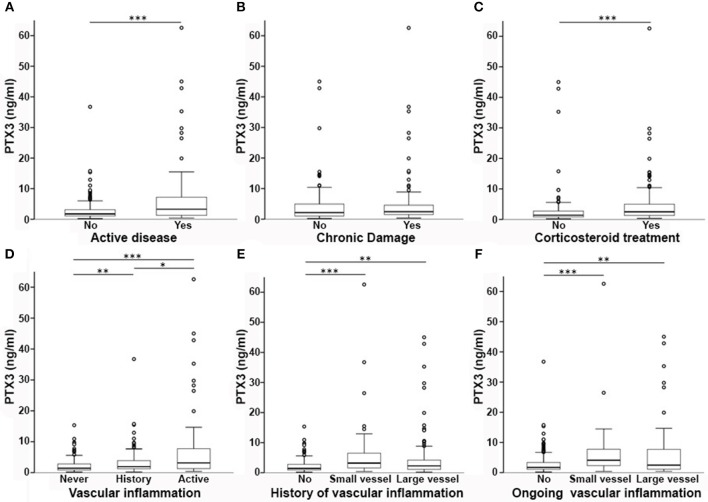
PTX3 levels and disease phenotypes. In this multi-panel graph, differences in PTX3 plasma levels among phenotype groups are highlighted. PTX3 levels were higher in patients with active disease **(A)**, but not with accrued irreversible damage **(B)**. PTX3 was also higher in patients on corticosteroids **(C)**. **(D)** Depicts the existing differences between patients with vs. without a history of vasculitis and between active or quiescent vasculitis at time of sampling. In **(E,F)** patients are stratified according to a history **(E)** or ongoing activity **(F)** of small- or large-vessel vasculitis associated with higher levels of PTX3 when compared to no vasculitis. ^*^p < 0.05; ^**^p < 0.01; ^***^p < 0.001.

On univariate analysis in the general group of subjects who had been studied, PTX3 levels were higher in females than in males (Figure 2A) and did not correlate with disease duration (Table 2). Patients with recent onset disease (i.e., up to 6 months) had higher PTX3 levels compared to patients with longer disease duration (Figure 2B). PTX3 levels positively correlated with Z-activity (Rho = 0.181, *p* = 0.016, *n* = 176 Figure 3A), with acute phase reactants (ESR and CRP, Rho = 0.229 and Rho = 0.128, *p* < 0.001 and *p* = 0.035, *n* = 261 and *n* = 269, respectively) and with steroid dose (Rho = 0.198, *p* = 0.001, *n* = 259; Table 1 and Figure 3B). On the contrary, PTX3 levels did not correlate with Z-damage (Rho = −0.006, *p* = 0.930). PTX3 levels were higher in patients with active disease as compared to those with quiescent disease (3.28, IQR 1.27–7.21, *n* = 123 vs. 1.74, IQR: 1.07–3.16, *n* = 222), while similar levels were observed in patients with or without organ damage. Accordingly, PTX3 levels were higher in patients with active disease and on corticosteroid treatment, but did not differ between patients with or without chronic damage (Figures 4A–C).

**Table 2:** Correlations among PTX3 plasma levels and clinical features at univariate analysis.

**Variable**	**Spearman's Rho**	***p***
Age (years)	NS	NS
Age at disease onset (years)	NS	NS
Z-score (activity)	0.181	0.016
Z-score (damage)	NS	NS
ESR (mm/h)	0.229	0.0002
CRP (mg/l)	0.128	0.035
Prednisone dose (mg)	0.198	0.001

### PTX3 Reflects Vascular Inflammation

Subjects with previous vasculitic manifestations had higher PTX3 levels than those without (2.3 ng/ml, IQR = 1.26–4.83, *n* = 220 vs. 1.43 ng/ml, IQR = 0.88–2.81, *n* = 146; *p* < 0.001) and patients with active vasculitis had higher levels of PTX3 compared to patients without evidence of vasculitis at time of sampling (3.15 ng/ml, IQR = 1.31–7.75, *n* = 85 vs. 1.72 ng/ml, IQR = 1.08–3.37, *n* = 278; *p* < 0.001 Figure 4D). PTX3 plasma levels were higher either in patients with a history of small-vessel inflammation (3.21 ng/ml, IQR = 1.57–6.58, *n* = 70) or large vessel inflammation (2.27 ng/ml, IQR 1.12–4.29, *n* = 138) as compared to those without history of vasculitic features (1.43 ng/ml, IQR = 0.88–2.81, *n* = 157; *p* < 0.001 and *p* = 0.003, respectively, Figure 4E). Moreover, active small-vessel inflammation (4.10 ng/ml, IQR = 2.16-8.09, *n* = 21) or active large-vessel inflammation (2.49 ng/ml, IQR = 1.10–7.95, *n* = 64) identified two subsets of patients with higher PTX3 plasma levels than patients without active vascular inflammation (1.72 ng/ml, IQR = 1.09–3.39, *n* = 281; *p* < 0.001 for both tests, Figure 4F).

### PTX3 and Disease-Specific Clinical Features

Both in SLE and AAV, pure vasculitic manifestations are not detectable in all cases. Analysis of the patients with AAV revealed a positive correlation of PTX3 and CRP levels (rho = 0.362; *p* = 0.005, *n* = 58), ESR (rho = 0.272; *p* = 0.037, *n* = 59) and ANCA titres at blood sampling (rho = 0.266; p = 0.049, *n* = 55). PTX3 also correlated with disease activity as measured by BVAS in patients with GPA and MPA (rho = 0.362; *p* = 0.014, *n* = 46). PTX3 levels were higher in patients with exclusive vasculitic manifestations (3.51 ng/ml, IQR = 2.00–7.92, *n* = 16 vs. 1.83 ng/ml, IQR = 1.28–2.97 in patients with concomitant granulomatous lesions, *n* = 47; *p* = 0.011) and lower in patients with ear-nose-throat involvement (1.84 ng/ml, IQR = 1.32–2.97, *n* = 43 vs. 3.37 ng/ml, IQR = 1.74–7.92, *n* = 20; *p* = 0.039).

PTX3 levels of patients with SLE correlated with disease activity as assessed by SLEDAI-2K in the whole group of patients (rho = 0.361; *p* = 0.007, *n* = 55) and in those who were off corticosteroids (*p* < 0.001, *n* = 19; Table 3), but not in patients receiving prednisone. In the latter patients, a positive correlation was observed between PTX3 levels and prednisone dose (rho = 0.198; *p* = 0.001, *n* = 36). Patients with >1 moderately-to-highly active (A, B) BILAG domain had significantly higher PTX3 levels than those with more limited disease activity extent (7.23 ng/ml, IQR = 5.51–9.58, *n* = 9 vs. 4.29 ng/ml. IQR = 3.09–6.34, *n* = 46; *p* = 0.041). PTX3 also directly correlated with PGA (rho = 0.383; *p* = 0.004, *n* = 55) and inversely with patient-reported VAS (rho = −0.331; *p* = 0.013, *n* = 55) and C4 levels (rho = −0.458; *p* = 0.001, *n* = 51). There was no significant correlation with age, disease duration or with CRP concentration or anti-DNA antibodies titres (Table 3). CRP was higher in patients with >1 A/B BILAG domain (*p* = 0.004), but its concentration did not correlate with SLEDAI-2K or prednisone dose. SLE patients with active disease tended to have higher levels of PTX3 compared to patients with inactive disease. This trend was more evident in patients with past or current evidence of vascular inflammation (Supplementary Figure 1).

**Table 3:** Correlations among PTX3 levels and clinical variables in patients with SLE.

	**Correlation with PTX3**	
	**All patients**	**Patients on PDN**
Age	No	No
Disease duration	No	No
SLEDAI-2K	Yes	No
PGA	Yes	No
Patient's VAS	Yes (inverse)	No
Erythrocyte sedimentation rate	No	No
CRP levels	No	No
C3 levels	No	No
C4 levels	Yes (inverse)	No
Anti-DNA titres	No	No
PDN dose	No	Yes

### PTX3 Levels Reflect Small- and Large-Vessel Inflammation at Multivariate Analysis

We performed two multivariate linear regressions of PTX3 plasma levels with a stepwise backward approach. The first regression included sex, diagnosis, disease activity, history and activity of small-vessel inflammation, history, and activity of large-vessel inflammation, and steroid therapy. The stepwise algorithm resulted in a model including disease activity (B = 3.162, Std.Err = 0.738, p < 0.001) and activity of small vessel inflammation (B = 4.200, Std.Err = 1.579, *p* = 0.008). In a second iteration, disease activity was excluded due to high colinearity with the other variables. The final regression model (Table 4) included activity of small vessel inflammation (B = 6.706, Std.Err = 1.473, *p* < 0.001), activity of large vessel inflammation (B = 4.269, Std.Err = 0.844, *p* = 0.008), and diagnosis (B = 0.243, Std.Err = 0.121, *p* = 0.046).

**Table 4:** Multivariate linear model of PTX3 levels.

	**B**	**Std. error**	***p*-value**
Active small-vessel inflammation	6.706	1.473	<0.001
Active large-vessel inflammation	4.269	0.844	0.008
Diagnosis	0.243	0.121	0.046
History of small-vessel inflammation	1.148	1.122	0.307
History of large-vessel inflammation	0.607	0.862	0.482
Sex	−0.354	0.750	0.637
Steroid therapy	−0.260	0.828	0.754

*R^2^ of the model including the significant variables: 0.122*.

## Discussion

In this brief report, we present a multi-disease comparison of PTX3 plasma profile in patients with various systemic autoimmune and inflammatory conditions. In line with previous evidence from other groups and us (20, 21, 23, 24, 26, 44–46), we observed that PTX3 levels rise in acutely inflamed patients. Accordingly, PTX3 levels are higher in patients with recent onset disease [see also (23, 47)] and correlated with disease activity and with conventional inflammatory markers such as ESR and CRP. However, PTX3 blood levels do not merely reflect systemic inflammation, and indeed they failed to increase in conditions such as RA and PMR. The relative lack of PTX3 increase well-agrees with the role of PTX3 as a tissue-generated signal: the inflamed *synoviae* possibly represent the preferential site of PTX3 generation and it has been reported that PTX3 assessment in the synovial fluid might indeed be more informative (27, 48).

Clinically overt vascular inflammation involving small or large vessels was associated with elevated PTX3 levels. Identification of reliable biomarkers for vascular inflammation assessment constitutes a significant unmet need in current Rheumatology practice (49–55). The present study supports the contention that vascular inflammation is a major driver of PTX3 elevation (20, 23, 56).

Patients with SLE had the highest PTX3 levels. PTX3 correlated with active SLE as estimated by the number of high-score BILAG domains. Aberrant presentation of autoantigens due to non-physiological release of PTX3 (13, 28, 57) could be involved, as indicated by the protective role of anti-PTX3 antibodies in SLE (58, 59). Corticosteroids are major inducers of PTX3 at a systemic level (60) and can constitute an additional modulatory variable in this setting. In particular, systemic administration of corticosteroid drugs or exposure to higher endogenous glucocorticoid levels cause an overall rise in blood PTX3 levels. Nonetheless, glucocorticoids have divergent effects on different cell types as they dampen PTX3 expression in monocyte-derived dendritic cells, but significantly induce PTX3 in endothelial cells and fibroblasts (60). Consistently, corticosteroids also exert distinct biological effects over different pathogenic backgrounds (61). Alternatively, smoldering vascular inflammation might be advocated as a potential explanation for plasma PTX3 elevation in patients with SLE and for conflicting results in the literature regarding associations with clinically overt vasculitis (26, 62–65).

Single tissue/organ-limited inflammatory events not involving the vascular bed might not represent effective stimuli for PTX3 elevation in the circulating blood. In line with this view, relatively low PTX3 concentrations were found in plasma from patients with RA, PMR and AAV without vasculitic features in this and other studies (20, 44). Neutrophil, endothelial cells and vessel-residing mononuclear cells can all concur to PTX3 release in the circulating blood during acute and chronic vascular injury (8, 11). PTX3 might be part of a protective response to the extension and exacerbation of organ damage due to post-ischemic inflammation (21, 29). However, PTX3 can also promote vascular injury under septic conditions (66). Furthermore, as a constituent of the antimicrobial array embedded in neutrophil extracellular traps (NETs) (10), PTX3 can concur to NETs-related immunothrombosis (58, 67) and similarly to neutrophil myeloperoxidase and proteinase-3 (which are also enclosed in NETs), promote the generation of pathogenic antibodies (68). Anti-PTX3 antibodies have been proposed represent atypical ANCA and, in contrast to SLE, might correlate with disease activity in patients with AAV (69).

This study has limitations. Systemic diseases without primary vessel inflammation are relatively underrepresented, which warrants caution in the interpretation of PTX3 dynamics in these settings. In addition, this study only explored the clinical relevance of PTX3 as a biomarker of vascular inflammation, without any deeper insight into the pathogenic drivers of this phenotype. Further mechanistic studies are thus needed to address this issue and possibly refine our knowledge on potential applications of PTX3 in diagnostics and therapy. To this purpose, dissecting the role of glucocorticoids as confounding factors for PTX3 expression would be of particular relevance, due to the widespread use of corticosteroid drugs in immune-mediated diseases.

Taken together, these data suggest that PTX3 could be implicated in multiple distinct pathophysiological events causing and maintaining inflammation in immune-mediated diseases. From a diagnostic point of view, PTX3 elevation in the circulating blood marks the occurrence of inflammatory events in blood vessels and might find a specific niche in clinical practice as a tool to identify vasculitic subsets among patients with autoimmune diseases.

## Ethics Statement

The study was performed under the Autoimmuno-Mol protocol, approved by the Ethics Committee of the San Raffaele Institute, Milan, Italy; reference number 2/2013/INT.

## Author Contributions

AAM, PR-Q, GR, and ET designed the study. MaB, GR, ET, MiB, SS, MD, and RD collected clinical data. RL performed PTX3 evaluation. AM supervised the laboratory analysis and provided critical intellectual contribution to the study design and implementation. GR and ET analyzed clinical and laboratory data. GR, ET, and AAM drafted and revised the manuscript. The final version of the manuscript was approved by all authors.

### Conflict of Interest Statement

The authors declare that the research was conducted in the absence of any commercial or financial relationships that could be construed as a potential conflict of interest.

## Supplementary Material

Supplementary Figure 1 PTX3 levels in SLE. Boxplots depicting the existing differences in PTX3 plasma levels among patients with SLE stratified by disease activity, history of vascular inflammation and vasculitic activity. Patients with active disease not comprising any vascular involvement (V.I.) and patients with active vascular involvement in SLE showed a trend toward higher PTX3 levels compared to patients with clinically quiescent disease.

## Abbreviations

AAV: ANCA-associated vasculitis
ACR: American College of Rheumatology
ANCA: anti-neutrophil cytoplasmic antibodies
BILAG: British Isles Lupus Assessment Group
BVAS: Birmingham Vasculitis Activity Score
CRP: C-reactive protein
DAS-28, 28-joint disease activity score EGPA: eosinophilic granulomatosis with polyangiitis
ESR: erythrocyte sedimentation rate
GPA: granulomatosis with polyangiitis
HC: healthy controls
ITAS: Indian Takayasu Activity Score
MPA: microscopic polyangiitis
PDN: prednisone
PGA: Physician's global assessment scale
PMR: polymyalgia rheumatica
PTX3: pentraxin-3
RA: rheumatoid arthritis
SDI: SLICC/ACR damage index
SLE: systemic lupus erythematosus
SLEDAI: SLE disease activity index
SLICC: SLE international collaborating clinics
TA: Takayasu's arteritis
TADS: TA Damage Score
VAS: visual analog scale
VDI: vasculitis damage index.
