# Corrigendum: PTX3 Intercepts Vascular Inflammation in Systemic Immune-Mediated Diseases

**DOI:** 10.3389/fimmu.2019.01755

**Published:** 2019-07-24

**Authors:** Giuseppe A. Ramirez, Patrizia Rovere-Querini, Miriam Blasi, Silvia Sartorelli, Maria Chiara Di Chio, Mattia Baldini, Rebecca De Lorenzo, Enrica P. Bozzolo, Roberto Leone, Alberto Mantovani, Angelo A. Manfredi, Enrico Tombetti

**Affiliations:** ^1^Università Vita-Salute San Raffaele, Milan, Italy; ^2^Unit of Immunology, Rheumatology, Allergy and Rare Diseases, IRCCS Ospedale San Raffaele, Milan, Italy; ^3^Division of Immunology, Transplantation and Infectious Immunity, IRCCS Ospedale San Raffaele, Milan, Italy; ^4^Humanitas Research Center - IRCCS, Rozzano, Italy; ^5^Humanitas University, Rozzano, Italy; ^6^The William Harvey Research Institute, Queen Mary University of London, London, United Kingdom

**Keywords:** PTX3, autoimmunity, lupus, rheumatoid arthritis, Takayasu arteritis, giant cell arteritis, ANCA associated small vessel vasculitis, intravascular immunity

In the original article, we neglected to include the funder **“**Cluster Alisei (MEDINTECH CTN01_00177_962865)” to Alberto Mantovani.

A correction has therefore been made to the **Funding** statement:

“This work was supported by grants from the MIUR (2015MGBEM2_002), and the Ministero della Salute (RF_2013_02358715) to AAM as well as the PR-Q and European Research Council (ERC – No 69415), Fondazione Cariplo (Contract no. 2015-0564) and Cluster Alisei (MEDINTECH CTN01_00177_962865) to AM.”

The authors apologize for this error and state that this does not change the scientific conclusions of the article in any way. The original article has been updated.

