# Supplementary figures and images for: PTX3 Intercepts Vascular Inflammation in Systemic Immune-Mediated Diseases
Source: Front Immunol. 2019 May 29;10:1135. doi: 10.3389/fimmu.2019.01135 (PMC6548810; doi:10.3389/fimmu.2019.01135)

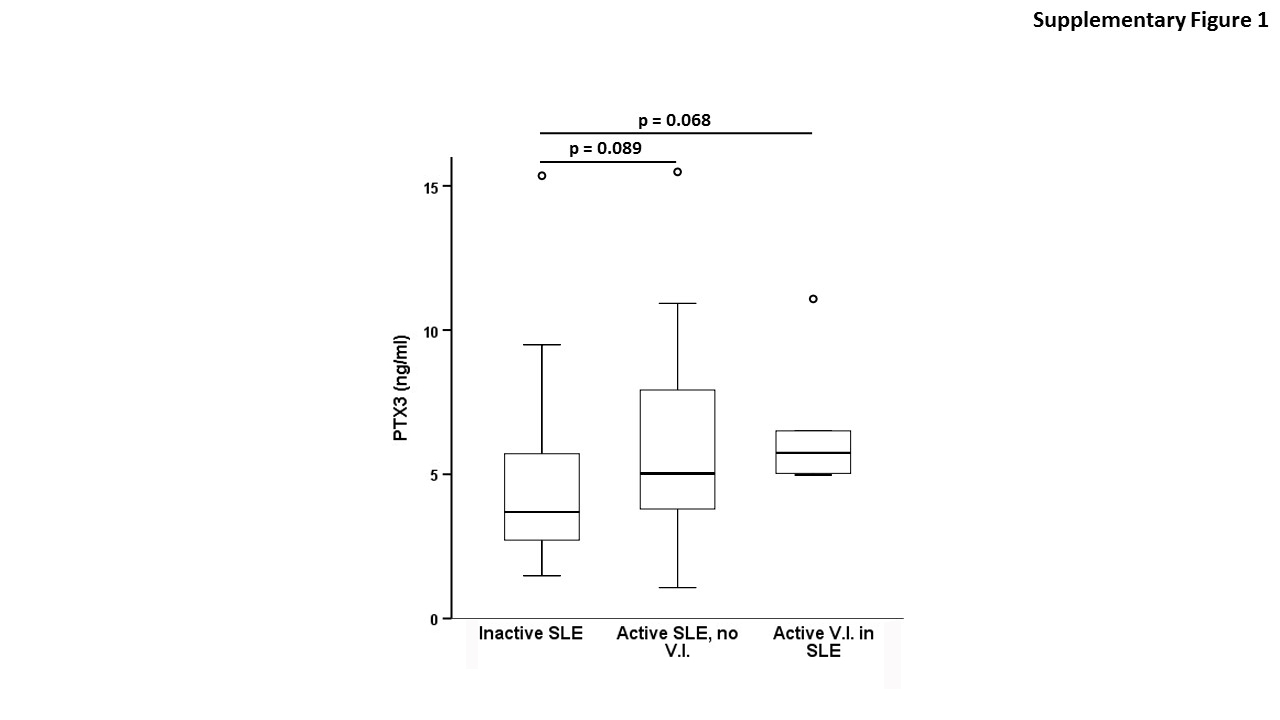

Supplement: Supplementary Figure 1 — PTX3 levels in SLE. Boxplots depicting the existing differences in PTX3 plasma levels among patients with SLE stratified by disease activity, history of vascular inflammation and vasculitic activity. Patients with active disease not comprising any vascular involvement (V.I.) and patients with active vascular involvement in SLE showed a trend toward higher PTX3 levels compared to patients with clinically quiescent disease. [file Image_1.JPEG]
